# Can prevalence data help us identify developmental language disorder?

**DOI:** 10.1016/j.lanwpc.2023.100714

**Published:** 2023-02-16

**Authors:** Jelena Kuvac Kraljevic

**Affiliations:** University of Zagreb Faculty of Education and Rehabilitation Sciences, Croatia

In 2017, a group of leading researchers and clinicians established new criteria for identifying developmental language disorder (DLD). That has broadened the phenotype of this condition mostly by decreasing the cut-off for nonverbal intelligence^1^ and it directly impacted the question of the DLD prevalence rate. Can we still rely on data provided by Bruce Tomblin and colleagues^2^ in 1997, who assumed a prevalence rate of 7.4%? The answer was very quickly provided by Norbury and colleagues^3^ who, applying the newly established diagnostic criteria, confirmed the prevalence of language disorder in five-year-old children in the United Kingdom to be 7.58%. Using this prevalence, Norbury and colleagues concluded that approximately two children in every class of 30 children are affected by a language disorder severe enough to impede academic progress. This study raises a new question, namely, whether this estimated prevalence rate is universal for all languages in terms of language typology and orthography or varies considerably under the influence of these linguistic factors.

The study by Wu and colleagues contributed to finding an answer by examining the prevalence of DLD in a sample of Mandarin-speaking children at the age of 5 and 6. Starting from a population-based survey with a cluster random sampling design in Shanghai, they finally recruited 1082 children who were approached for an onsite evaluation of their language skills. The results indicated 8.5% of children with DLD which is similar to the results obtained in Norbury et al.'s study.^4^

Why is prevalence data so important? DLD is considered a ‘hidden’ disorder because children with DLD are initially indistinguishable from typical peers, which impacts the timely identification and treatment of this disorder. If the disorder is subtle, and therefore less known to the public because the public “cannot see it easily” (such as stuttering), then more different approaches to identifying children with DLD should be developed and taken. One of the approaches can be the prevalence rate. In the case of DLD, the knowledge of prevalence can be valuable for several reasons: if we know the prevalence, we have initial information about how many individuals with language difficulties should be identified on purpose in our society and be treated – this implies a prescribed and well-developed screening procedure; if the prevalence is high, as is the case with DLD, then the prevalence data may be a trigger to raise public awareness of this particular condition - this means that information about prevalence needs to be disseminated and promoted more frequently through the media and academia; if the public is more sensitive on DLD, changes in education and health policy may be more rapid and widespread; and finally, greater public awareness may be the trigger for greater investment in research at the academic level, which to date has been too small and insufficient for DLD.^5^ This example illustrates how the entire system of access to a clinical population has the effect of connected vessels.

In addition to prevalence data, the study by Wu et al. confirmed a stronger association of DLD with other concurrent difficulties such as social-emotional difficulties and poor school readiness, as well as two risk factors - a lack of diversity in parent–child interactions and lower kindergarten attainment. The identification of risk factors is directly related to the design of prevention programs. Prevention is an extremely important investment in the community and its population, as it prevents the development of many negative factors and has a great return on investment in health and education in the long term. If we know the risk factors and act accordingly, we may not be able to prevent the occurrence of DLD, which has a genetic and biological basis, but we can certainly influence a number of outcomes of DLD especially those that have onset on the adolescent period, such as psycho-emotional difficulties. As a large body of literature emphasizes, DLD represents a social burden, because early language problems are highly associate with externalizing (e.g., physical aggression) and internalizing (e.g., anxiety) psychological problems.^6^

The study by Wu et al. suggests that high-quality, diverse interactions with parents, teachers, and peers may be important protective factors for DLD. This implies that coordinated efforts are needed across sectors to identify populations affected by DLD earlier, more reliably, and to take appropriate action consequently. Finally, this study provides important epidemiologic data on DLD that directly impact many aspects of education, social care, and well-being for this large group of children.

## Declaration of interests

The author have no known conflict of interest to disclose.
